# The Veterans Health Administration Reproductive Mental Health Consultation Program: an Innovation to Improve Access to Specialty Care

**DOI:** 10.1007/s11606-022-07583-5

**Published:** 2022-08-30

**Authors:** Laura J. Miller, Sandy Rowlands, Laura Esposito, Margaret Altemus, Jennifer L. Strauss

**Affiliations:** 1 Women’s Mental Health, Office of Mental Health and Suicide Prevention, Veterans Health Administration, Washington DC, USA; 2 Loyola University Stritch School of Medicine, Maywood, IL USA; 3 Edward Hines Jr VA Hospital, Hines, IL USA; 4 VA Connecticut Healthcare System, West Haven, CT USA; 5 Yale School of Medicine, New Haven, CT USA; 6 Duke School of Medicine, Durham, NC USA

## INTRODUCTION

Reproductive mental health needs are prevalent, under-recognized, and under-treated throughout the United States (US).^1^ This especially affects the Veterans Health Administration (VHA) as the population of US veterans who are women continues to increase. Between 1980 and 2018, the percent of women veterans more than doubled, from approximately 4 to 9%, and is projected to increase to 17% by 2040. More than 75% of women veterans are of reproductive age, less than 45 years old.^2^

This has led to a sizeable increase in reproductive mental health (RMH) needs among women veterans. Among women receiving health care through VHA, the number who gave birth increased more than 14-fold from the year 2000 to 2015.^3^ Based on multisite and national data, it is estimated that among pregnant women receiving VHA care, 28% have clinically significant depression^4^ (more than double the estimated 12% prevalence of perinatal depression in the general population)^5^ and 12% have post-traumatic stress disorder (PTSD).^6^ Untreated antenatal depressive and/or PTSD symptoms are associated with increased risk of obstetric complications and adverse effects on offspring.^7–9^

In addition to perinatal mental health concerns, other reproductive-related conditions require specialized clinical knowledge and interventions. For example, premenstrual dysphoric disorder requires specific tools and processes for diagnosis, responds to specific psychotropic agents, and can be treated with unique dosing strategies and psychotherapy adaptations.^10^ Similarly, perimenopausal depression requires unique diagnostic strategies and responds best to multimodal, targeted interventions.^11^

Throughout the US, the need for RMH services exceeds available expertise, in part due to a dearth of RMH training.^12^ The challenge of ensuring access to specialized RMH knowledge is amplified in VHA, where a small proportion of patients are women and 44% of those women are diagnosed with a mental health condition (Greenberg 2020).^13^ VHA is also among the largest health care systems in the US, serving remote rural as well as urban populations; an effective system of access to RMH specialty knowledge needs to reach all veterans while using resources efficiently.

In circumstances of substantial clinical need but limited locally available expertise, a consultation program can greatly expand access to care. Perinatal mental health consultation programs have attempted to fill this gap, including three state-based and one national program^14–17^. These models expanded access but have limited applicability for VHA. All were perinatal only; none included the full range of RMH concerns. Other limitations included being geared to limited groups of clinicians (e.g., obstetricians), solely addressing psychopharmacology questions, or lacking adequate quality control.

Learning from the strengths and limitations of these programs and adapting to the needs of VHA, we developed a national RMH Consultation Program to ensure access to RMH expertise across VHA. The VA RMH Consult Program differs from others in several ways:

- It addresses a wide range of women’s RMH concerns including but not limited to perinatal mental health. This is particularly important for a health care system which serves mainly men.
- Replies are in writing to ensure accuracy and retention and promotes sharing replies with other health care team members. This confers medicolegal protection by stating the purpose and limitations of the consultation, and documenting replies.
- It is a multidisciplinary team of experts available to all types of clinicians and not limited to pharmacologic questions.
- Multiple team members review each consult reply and come to consensus, offering more quality control than in services where only one consultant replies.

We conducted an initial evaluation of the VA RMH Consult Program by measuring utilization patterns and satisfaction ratings for the first 200 consultations. This initial evaluation focused on the feasibility, effectiveness, and scalability of the program.

## SETTINGS AND PARTICIPANTS

The VHA RMH Consultation Program is virtual, accessed by email. It was piloted at 12 geographically diverse VHA health care systems in 2019 and implemented nationally, across all VHA health care systems, in 2020.

Program evaluation data are based on the first 200 consultations, received between 5/20/19 and 6/10/21 from 122 unique consultees. Among these, 188 were initial consultations; 12 were follow-up consultations for the same cases. Among the 122 consultees, 93 placed a single consult, 12 placed 2 consults, 6 placed 3 consults, 4 placed 4 consults, 3 placed 5 consults, 2 placed 6 consults, and 2 placed 7 consults.

## PROGRAM DESCRIPTION

This program was developed to fill a need identified by demographic data and through VHA’s network of Women’s Mental Health Champions (WMHCs). Each VHA medical center has at least one WMHC, who serves as a local point of contact for advancing WMH services and communicating WMH needs. A survey of WMHCs revealed that few VHA medical centers had sufficient resources to address RMH concerns.

The consultation team consists of two psychiatrists, a clinical pharmacy specialist, and a social worker, all with extensive subject matter expertise, experience, and training in RMH. The team also includes multidisciplinary trainees; this expands capability while providing mentoring.

The VA RMH Consultation Program accepts requests about premenstrual, perinatal, and perimenopausal mental health concerns. It also covers mental health questions related to contraception, infertility, perinatal loss, breast and gynecologic cancers, and gynecologic comorbidities.

Clinicians within any discipline, at any VA health care facility, can email consultation requests to a dedicated address. Queries contain no personal identifying information about patients. A brief request form, with a sample query, helps consultees provide relevant information. The team social worker reviews the query and asks for further information if needed. A team psychiatrist reviews relevant research and drafts a reply. Replies typically include 3–6 pages of evidence-based explanations for relevant clinical issues, including but not limited to those explicitly asked. For example, if the query is about pharmacotherapy, the reply may also include information about evidence-based nonpharmacologic interventions. The draft ends with a “key points” summary. This draft is reviewed by other team members, who comment and edit. When consensus is reached, the reply is emailed to the consultee. The consultee is encouraged to share the reply with health care team members and to place it in the medical record.

In developing this service, the primary barriers anticipated were making clinicians aware of its availability, and lack of detection of RMH issues. To address the first barrier, information about the program was disseminated via the WMHC network. The program was presented on numerous national calls for relevant VHA communities of practice, such as psychiatrists, maternity care coordinators, and women’s health primary care providers. To address the second barrier, VHA developed and delivered a 12-h training session on RMH, available virtually to all VHA clinicians.

## PROGRAM EVALUATION

This initial evaluation focused on the feasibility, effectiveness, and scalability of the program. Consultees were asked to complete brief feedback forms that were emailed along with consult replies. Feedback was received from 82 consultees. As feedback was voluntary, we prioritized not placing additional time burdens on clinicians and opted not to make second requests of non-responders.

### Feasibility

Objective #1: Prompt replies — measured by tracking response times and asking users whether replies were prompt enough (yes/no). The average response time was 1.7 business days (range 7; standard deviation 1.3); 100% of respondents said the replies were prompt enough.

Objective #2: Serving all geographic regions — measured by tracking responses by Veterans Integrated Service Networks (VISNs). Requests came from all but one of VA’s 18 VISNs. There was a median of 10 requests per VISN, with a range of 0–30.

Objective #3: Serving multidisciplinary mental health clinicians, with availability to other types of clinicians — measured by tracking users’ clinical disciplines. Table 1 shows the clinical disciplines of consultees. Most users were mental health providers, most often psychiatrists. Clinical pharmacists, primary care physicians, and gynecologists were not the primary intended users but were welcome to use the service; this is reflected in their smaller presence.

**Table 1:** Percent of Unique Consultees by Clinical Discipline (*N*=123)

Clinician discipline	Number and percent
Psychiatrist	69 (56.1%)
Advanced practice nurse	14 (11.4%)
Psychologist	19 (15.4%)
Clinical pharmacist	9 (7.3%)
Social worker	6 (4.9%)
Primary care physician	3 (2.4%)
Certified physician assistant	2 (1.6%)
Gynecologist	1 (0.8%)

Objective #4: Responding to a wide array of RMH concerns — measured by tracking type of RMH query. Table 2 shows the primary topics for which consultations were requested. The requests span the full array of topics addressed by this service, with most questions related to perinatal concerns.

**Table 2:** Topic Requests for Reproductive Mental Health Consultation (*N*=200)

Primary reproductive mental health topic	Number and percent
Perinatal (pregnancy and postpartum)	117 (58.5%)
Preconception planning	49 (24.5%)
Perimenopause	13 (6.5%)
Premenstrual mental health symptoms	11 (5.5%)
Breast cancer	2 (1.0%)
Breastfeeding and weaning	2 (1.0%)
Hysterectomy	2 (1.0%)
Post-menopausal vasomotor symptoms	1 (0.5%)
Infertility	1 (0.5%)
Mental health effects of contraceptives	1 (0.5%)
Sexual functioning	1 (0.5%)

Objective #5: Summarizing complex concepts in a way that is easy for busy clinicians of multiple disciplines to understand — measured by surveying users (yes/no). Among respondents, 81 (98.8%) said the reply was easy to understand.

### Effectiveness

Since cases have a variety of diagnoses and reproductive concerns, no single set of clinical outcome measures is applicable. Although change in clinical practice is desirable in some cases, in other cases consultations reinforce clinicians’ treatment plans when clinicians feel uncertain. For this reason, we focused the program evaluation on a universally applicable concept: whether clinicians found the consultations useful. To ascertain whether this was a *necessary* resource, we asked clinicians to compare it to other available local and online resources.

Consultees were asked to rate usefulness on a 4-point scale (not useful, a little useful, moderately useful, and very useful). Among the 82 respondents, 78 (95.1%) rated replies as very useful; the other 4 (4.9%) rated them as moderately useful.

Consultees were asked to compare usefulness to online and local resources on a 4-point scale (less helpful, about the same, more helpful, and much more helpful). Regarding online resources, 75/82 (91.5%) rated the consult replies much more helpful, 6/82 (7.3%) rated them more helpful, and 1/82 (1.2%) rated them about the same. Only 74 respondents replied to the question about local resources; some non-respondents were unaware of any local resources. Among the 74 respondents, 63 (85.1%) found the national consults much more helpful; 11 (14.9%) found the national consults more helpful.

Qualitative comments from consultees were overwhelmingly positive. These suggested additional benefits, such as improved confidence, time saving, ability to teach patients, and ability to weigh risks and benefits. Examples include:

- “The response was prompt, thorough and with the most up to date information and data. I feel much more confident and reassured in treating my pregnant patient.”
- “The suggestions were detailed, specific and action-oriented.”
- “I feel like the consults help teach me so I can apply it to future patients.”
- “I found the thorough weighing of risks and benefits helpful.”
- “It personalized the response to the care of the veteran.”
- “Much easier to understand to relay to the veteran.”
- “Provides an excellent summary of evidence-based literature allowing provider to focus on other aspects of the patient’s care.”

### Scalability

To maximize scalability, response times were estimated to promote efficient practices. During the pilot phase, drafting a response took approximately 3–4 h, including review of relevant literature. Responses are logged by subject, so information remains consistent in subsequent replies until updates are needed. This reduced estimated time to draft a response to 0.5–1.5 h. The estimated time for team members to review and edit a draft is 10–20 min per person.

## DISCUSSION AND FUTURE DIRECTIONS

As the proportion of veterans who are women grows, meeting the RMH needs of this subpopulation is especially important, yet challenging.

Implementing the RMH Consultation Program is part of addressing this challenge. While especially suited to VHA, this model could be adapted for use in other health networks.

Preliminary evaluation data show feasibility and high user satisfaction, with the caveat that data are from voluntary respondents only. The eventual scope of need is unknown; preliminary data suggest scalability. The program’s limitations reflect the anticipated barriers. While nearly all geographic regions have utilized this service, the wide range of usage suggests underutilization in some areas. More widespread dissemination of information about this service is a goal for the next phase of development of this program. The paucity of consult requests about non-perinatal topics highlights a systemic need to ensure awareness and detection of these conditions. In a pilot study^18^ of women receiving mental health evaluations at a VHA Women’s Health Clinic, 43.3% of participants screened positive for premenstrual emotional problems and 31.2% screened positive for perimenopausal emotional problems, suggesting that the number of women veterans who might benefit from consultation about these conditions likely exceeds current request volume. This can inform future efforts to expand RMH awareness, clinician training, and patient screening throughout VHA.
